# Exclusion of Superinfection or Enhancement of Superinfection in Pestiviruses—APPV Infection Is Not Dependent on ADAM17

**DOI:** 10.3390/v16121834

**Published:** 2024-11-26

**Authors:** Francesco Geranio, Sebastian Affeldt, Angelika Cechini, Sandra Barth, Carina M. Reuscher, Christiane Riedel, Till Rümenapf, Benjamin Lamp

**Affiliations:** 1 Institute of Virology, Faculty of Veterinary Medicine, Justus-Liebig-University Giessen, Schubertstrasse 81, 35392 Giessen, Germany; francesco.geranio@uni-marburg.de (F.G.); sebastian.affeldt@vetmed.uni-giessen.de (S.A.); angelika.cechini@lhl.hessen.de (A.C.); sandra.barth-2@vetmed.uni-giessen.de (S.B.);; 2 CIRI-Centre International de Recherche en Infectiologie, Université Lyon, Université Claude Bernard Lyon 1, Inserm, U1111, CNRS, UMR5308, ENS Lyon, 46 Allée d’Italie, 69007 Lyon, France; christiane.riedel@ens-lyon.fr; 3 Institute of Virology, Department of Pathobiology, University of Veterinary Medicine, 1210 Vienna, Austria; till.ruemenapf@vetmeduni.ac.at

**Keywords:** pestivirus, superinfection exclusion, CSFV, classical swine fever virus, BVDV, bovine viral diarrhea virus, APPV, atypical porcine pestivirus, BDV, border disease virus, Linda pestivirus, Bungowannah virus, ADAM17

## Abstract

Some viruses can suppress superinfections of their host cells by related or different virus species. The phenomenon of superinfection exclusion can be caused by inhibiting virus attachment, receptor binding and entry, by replication interference, or competition for host cell resources. Blocking attachment and entry not only prevents unproductive double infections but also stops newly produced virions from re-entering the cell post-exocytosis. In this study, we investigated the exclusion of superinfections between the different pestivirus species. Bovine and porcine cells pre-infected with non-cytopathogenic pestivirus strains were evaluated for susceptibility to subsequent superinfection using comparative titrations. Our findings revealed significant variation in exclusion potency depending on the pre- and superinfecting virus species, as well as the host cell species. Despite this variability, all tested classical pestivirus species reduced host cell susceptibility to subsequent infections, indicating a conserved entry mechanism. Unexpectedly, pre-infection with atypical porcine pestivirus (APPV) increased host cell susceptibility to classical pestiviruses. Further analysis showed that APPV can infect SK-6 cells independently of ADAM17, a critical attachment factor for the classical pestiviruses. These results indicate that APPV uses different binding and entry mechanisms than the other pestiviruses. The observed increase in the susceptibility of cells post-APPV infection warrants further investigation and could have practical implications, such as aiding challenging pestivirus isolation from diagnostic samples.

## 1. Introduction

The phenomenon of superinfection exclusion or viral interference has been described for different virus families and can be caused by different mechanisms. Inactivation of cellular receptors and transport molecules can be observed already in bacteria after T5 phage infection, where the receptor molecules on the bacterial surface are blocked by small lipoproteins [1]. Well-known examples from eukaryotic viruses include different retroviruses, vesicular stomatitis virus, Borna disease virus, alphaviruses, and pestiviruses. Individual mechanisms underlying superinfection exclusion have been linked to the different stages of the viral replication cycle. Interference may arise in attachment or receptor binding [2,3,4,5], endocytosis and cell membrane penetration [6,7,8], as well as early steps of genome replication [9,10,11,12]. However, these phenomena are mostly restricted to individual combinations of pre-infections and superinfections. For pestiviruses, a strong E2-mediated block of viral entry has been described using bovine viral diarrhea virus (BVDV) infection or the sole expression of the E2-ectodomain [13]. The exact molecular mechanisms responsible for this blockade are still unclear. A second line of interference was identified during early pestiviral replication steps, not involving structural protein expression [14]. Interestingly, these processes not only matter in cultured cells, but were also observed in vivo in case of classical swine fever virus (CSFV) double infections [15]. The pestiviral superinfection exclusion phenomenon is highly specific and can even be exploited for diagnostic purposes, such as the use of reporter viruses for the indirect identification and quantification of pestiviruses in diagnostic samples [16].

In this study, we aimed at characterizing the superinfection exclusion phenomenon between the different pestivirus species in more detail. The individual pestivirus species form the distinct genus *Pestivirus* within the family *Flaviviridae*. Typical representatives of this genus are economically important pathogens of cloven-hooved animals long-known as CSFV, BVDV-1 and BVDV-2, and border disease virus (BDV). More recent taxonomic adaptations have led to the introduction of proper species names (binary combinations of the genus name followed by a single specific epithet) such as *Pestivirus bovis* (BVDV-1)*, Pestivirus tauri* (BVDV-2)*, Pestivirus suis* (CSFV)*,* and *Pestivirus ovis* (BDV) [17]. In the recent past, several new species have been identified expanding the known host range of pestiviruses to cetaceans (*Pestivirus M*) [18], pangolins (*Pestivirus P*) [19], bats (*Pestivirus S*) [20], and rodents (*Pestivirus R* and *Pestivirus ratti*) [21,22]. However, additional pestivirus species have also been discovered and described in the typical ungulate hosts, such as HoBi-like pestiviruses (HoBi or BVDV-3, *Pestivirus brazilense*) [23], Bungowannah pestivirus (BuPV, *Pestivirus australiaense*) [24], lateral-shaking inducing neurodegenerative agent (Linda virus, *Pestivirus L*) [25], atypical porcine pestivirus (APPV, *Pestivirus scrofae*) [26], Tunisian sheep-like pestivirus (TSPV, *Pestivirus N*) [27], and, most recently, Italian ovine pestiviruses (IOPV, *Pestivirus O*) [28].

Pestiviruses are small, enveloped RNA viruses with common characteristic features. Their plus-stranded RNA genome of about 12 kb encodes a single open reading frame (ORF), which is flanked by 5′ and 3′ untranslated regions (UTR). An internal ribosomal entry site (IRES) element within the larger 5′-UTR mediates the translation of a huge polyprotein of about 3000 amino acids. This polyprotein is co- and post-translationally processed by cellular and viral proteases into four structural proteins (Core, E^rns^, E1, and E2) [29] and at least eight mature nonstructural proteins, namely N^pro^, p7, NS2, NS3, NS4A, NS4B, NS5A, and NS5B [30,31]. The virion envelope contains the glycoproteins E^rns^, E1, and E2 in homo- and heterodimeric states, which mediate attachment, receptor binding and endocytosis. A major viral protease, termed NS3, is a key replication enzyme, whose release is tightly regulated by a cellular co-factor, thereby coupling viral replication to the cellular metabolism [32]. The non-cytopathogenic (ncp) biotype of pestiviruses can persist in cell cultures without causing visible damage to their host cells. Ncp pestiviruses are the dominant biotype in nature and can induce persistent infections after diaplacental infections. Deregulation of the NS2-3 processing by mutation may result in damage to the host cell and subsequent apoptosis as seen in the cytopathogenic (cp) biotype of pestiviruses. Cytopathogenic strains of BVDV may arise in persistently infected (PI) calves. Continuous viral replication under the protection of immune tolerance favors mutation or recombination with cellular genes, such as the NS2 co-factor DNAJC14. Deregulated NS3 release by the NS2 autoprotease increases the replication levels, ultimately inducing lethal mucosal disease in these PI animals [33].

Much is known about the infection process of pestiviruses. Neutralizing antibodies against E^rns^ and E2 have been characterized underlining the importance of both glycoproteins during CSFV entry [34]. E^rns^ is an essential component of the virion and responsible for an attachment to the host cell surface via heparan sulfates—at least in cell culture-adapted strains of CSFV [35]. However, the E1 and E2 heterodimer is sufficient and essential for receptor binding and entry in case of BVDV [36]. Exchange of the E2s between BVDV and CSFV using reverse genetic systems showed that solely the identity of the E2 protein determines host cell tropism preferences [37]. The molecular structures of E^rns^ and E2 of BVDV have been solved using X-ray crystallography [38,39,40]. Different cellular attachment factors and receptor molecules have been proposed [41], of which CD46 [42,43,44,45] and the metalloprotease ADAM17 [46,47] were confirmed by independent groups in independent experiments. The absence of heparan sulfate or CD46 failed to completely block host cell infection in the case of BVDV and APPV [48]. The absence of ADAM17 completely eliminated BVDV permissiveness in CRIB-1 cells, a BVDV-resistant MDBK clone, underscoring ADAM17’s critical role as an attachment factor in the pestiviral infection cycle [47]. ADAM17, also termed tumor necrosis factor-α-converting enzyme (TACE), belongs to the “A disintegrin and metalloproteinases” (ADAMs) family also designated as “metalloproteases, disintegrins, cysteine-rich proteins” (MDCs) [49]. ADAMs are responsible for the processing and shedding of multiple transmembrane proteins. Yuan et al. showed a direct interaction of CSFV E2 with the protease domain of ADAM17, which was essential for virus entry and infection, but not species-specific for the porcine ADAM17 [46].

Here, we characterized superinfection exclusion between the different pestivirus species. Surprisingly, we found that APPV pre-infection increases the susceptibility of SK-6 cells to classical pestiviruses. We also discovered that APPV does not rely on the attachment factor ADAM17, which is crucial for the other pestiviruses. The independence from key host factors like DNAJC14 and ADAM17 may explain the absence of superinfection exclusion in APPV-infected cells and underlines that APPV is indeed an atypical pestivirus.

## 2. Materials and Methods

### 2.1. Cells and Viruses

The swine kidney cell line SK-6 [50], the bovine kidney cell line MDBK [51], and the human cell line 293T [52] were obtained from the cell bank of the Institute of Virology in Giessen, Germany. All cells were cultured in Dulbecco’s Modified Eagle Medium (DMEM; Thermo Fisher Scientific, Waltham, MA, USA) supplemented with 10% fetal bovine serum (FBS Gold; Bio&Sell, Feucht, Germany), 100 U/mL penicillin, and 100 µg/mL streptomycin (Thermo Fisher Scientific) and incubated at 37 °C in a 5% CO_2_ atmosphere. The chromatographically produced “FBS Gold” was used to exclude the possibility of contaminations with pestiviruses of bovine serum origin.

CSFV strain Alfort-Tuebingen [53], BDV-1 strain X818 [54], BVDV-2 strain 890 [55], BVDV-3 strain GI2012 [56], and APPV strain AUT-2016_C [57] were sourced from the virus collection at the Institute of Virology, Giessen. The dual reporter CSFV (cp-CSFV-mCherry) and the cell culture-adapted APPV clone were developed in the Institute of Virology in Gießen, as previously described [33,57]. BVDV-1b strain NCP7 was generously provided by E. Dubovi (Cornell University College of Veterinary Medicine; Ithaca, NY, USA) [58]. Pestivirus strain Giraffe-1 was obtained from D. J. Paton (Animal Health and Veterinary Laboratory Agency; Weybridge, UK) [59]. Bungowannah virus was a gift from Peter Kirkland (Elizabeth Macarthur Agricultural Institute; Menangle, NSW, Australia) [60]. Linda pestivirus was obtained from the virus collection at the Institute of Virology, University of Veterinary Medicine, Vienna, Austria [61].

### 2.2. Generation of Pre-Infected Cell Lines

Cultures of MDBK and SK-6 cells were pre-infected with different pestiviruses to analyze the effects on the efficiency of superinfection with other pestiviruses. For this purpose, 1 × 10^6^ cells were seeded in 6-well plates and infected with an MOI > 1. After three days, when the cells had formed a dense monolayer and medium nutrients were depleted, the cells were harvested by trypsinization (passage 1). Then, the cells were transferred to a 10 cm dish (passage 2). Another three days later, the cells were passaged again using a 1:10 split ratio (passage 3). At this stage, the cell lines were harvested and aliquoted for immunodetection of infection, cryopreservation, and further passage. As expected, certain virus–cell combinations failed to achieve sufficient infection or spread, with immunostaining showing either no infection or solely isolated infected cells. These virus–cell combinations were excluded from further analyses.

Since wild-type APPV strains do not spread in cell cultures, we opted to test a different approach for the generation of an APPV-pre-infected SK-6 cell line. The SK-6 cells (1 × 10^6^) were transfected via electroporation with 1 µg of synthetic RNA, which was generated using SP6 polymerase and our established reverse genetics system for APPV [57]. Briefly, the electroporation setup involved a 0.18 kV pulse, 950 µF capacitance, and 300 µL cell suspension in a 2 mm cuvette (Gene Pulser II; Bio-Rad, Hercules, CA, USA). Electroporation was highly efficient (~90%), and although APPV wild-type viruses show limited post-electroporation spread, complete infection was observed following passage 3. These pre-infected cell lines were then used in our superinfection experiments, in which different pestiviruses were titrated on the naïve and pre-infected cells.

### 2.3. Determination of Virus Titers and Viral Focus Size

Virus titers were determined as 50% tissue culture infectious dose (TCID_50_) [62] or as focus forming units (FFU). For this purpose, the host cells were first seeded in 96-well cell culture plates (Starlab, Hamburg, Germany) to allow monolayer formation. On the following day, serial virus dilutions were prepared in uncoated 96-well plates and pipetted into the individual wells. Then, the cells were incubated to allow infection and subsequent focus formation. After 48 h, the cells were fixed and analyzed by different immunofluorescence assays, as described below. The individual wells were inspected using a fluorescence microscope (Olympus IX70 fluorescence microscope; Olympus, Hamburg, Germany) before calculating TCID_50_ values using the Spearman–Kaerber method. FFUs were counted and a mean was calculated for the cell culture-adapted APPV, as the virus titer was low and the spread was slow. To analyze the spread of the viruses from cell to cell, the infected cells of ten randomly selected, separate foci were counted. A mean value as well as the standard deviation (SD) were calculated. Images of representative foci were taken with a monochromatic digital camera (DFC3000G; Leica, Wetzlar, Germany) for visualization purposes.

### 2.4. Indirect Immunofluorescence Assays

For immunofluorescence staining, cells were washed with phosphate buffered saline (PBS; Carl Roth, Karlsruhe, Germany) and fixed with 4% paraformaldehyde (PFA; Carl Roth) for 20 min at 4 °C. Again, the cells were washed with PBS and permeabilized with 0.5% Triton-X 100 in PBS for 5 min at room temperature (RT). After another washing step with PBS, the primary antibody was added in PBS with 0.01% Tween-20 (PBSt) and incubated with the cells for 1 h. Different established monoclonal antibodies (mAbs) with different species specificities were tested and used for the species-specific detection of pestiviral infections (Table 1). An anti V5-tag Mab was obtained from Novex (Life technologies, Carlsbad, CA, USA) for the detection of ADAM17-V5. After three washing steps with PBSt, Cy3 or FITC, conjugated goat anti-mouse IgG (Dianova, Hamburg, Germany) was added and incubated for an hour. Then, the cells were incubated with 1 µg/mL DAPI (Thermo Fisher Scientific) in PBSt for 5 min for nuclear counterstaining. The cells were finally washed twice with PBSt and the staining was evaluated using a fluorescence microscope and a digital camera.

### 2.5. Calculation of Superinfection Rates

The remaining infectivity of the superinfecting virus on pre-infected cells was assessed for each individual combination of pre-infecting and superinfecting virus by comparative titration experiments. Defined stocks of the viruses were titrated in three separated replicates side-by-side on uninfected (naïve) host cells and on pre-infected cultures of the same cell line. Using three independent experiments and three replicates each, a mean TCID_50_ value was calculated for each virus stock on the naïve cells and defined as 100%. The mean TCID_50_ values of the same virus stock measured on the pre-infected cell cultures were then set in relation to the titers on the naïve cells and quantified as a percentage rate of residual infectivity using the following formula:

Superinfection rate [%] = (mean TCID_50_ on pre-infected cells)/(mean TCID_50_ on naïve cells) × 100.

### 2.6. CRISPR/Cas9-Mediated Knockout of ADAM17 in SK-6 Cells

With the help of a design tool (http://crispor.tefor.net, accessed on 25 November 2022), a suitable single guide RNA (sgRNA) sequence for the CRISPR/Cas9-mediated knockout of ADAM17 was determined in the pig genome (NCBI GCF_000003025.6, Ssctofa11.1). The 20-nucleotide-long guide RNA sequence 5′-GCATTCGCCAGCACTCCGTA-3′ was then inserted into the plasmid pLentiCRISPRv2 [66] employing BSmBI digestion and the two annealed oligonucleotides ADAM17-guide_fwd (5′-CACCGCATTCGCCAGCACTCCGTA-3′ and _rev (5′-AAACTACGGAGTGCTGGCGAATGC-3′). After pseudoparticle production (see below) and transduction of SK-6 cells, single-cell clones were obtained by puromycin selection and serial dilution. The mRNA of the ADAM17 gene was amplified from the resulting knockout clones using RT-PCR and sequenced following T-vector cloning. Clone 1 (used in this study) showed a larger deletion of 65 bp in one allele (deletion: 5′-aaaagcttgattctctgctctcagactacgacatcctctctttatccagcattcgccagcactcc-3′) and the insertion of a cytosine (insertion in lower case: 5′-GCATTCGCCAGCACTCCcGTA-3′) in the other allele—both located at the desired cleavage site within the guide sequence. Since both mutations shift the reading frame shortly after the start codon, expression of ADAM17 could be reliably excluded. In order to achieve effective overexpression of the ADAM17 gene in the case of a possibly still active gene scissors, the guide sequence including the PAM was mutated in the expression vector for the ADAM17 knock-in (see below).

### 2.7. Recombinant Expression Vector for Porcine ADAM17

A recombination cloning strategy was applied for the generation of a retroviral expression vector for the porcine ADAM17 gene. Total RNA of SK-6 cells was prepared (RNeasy Kit; Qiagen, Hilden, Germany) and transcribed using oligo-dT and Omniscript reverse transcriptase (Qiagen). The mature ADAM17 coding mRNA was amplified with the oligonucleotides ADAM17_forw (5′-ATGAGGCAGTGTGCGCTCTTC-3′) and ADAM17_rev (5′-CTAGCACTCCGTTTCCTTGCTGTC-3′) via OneTaq DNA polymerase (NEB) and subcloned in a T-vector (pGEM-T; Promega, Madison, WI, USA). After sequencing, the gene was inserted in a pEGIP-Blast vector providing a signal peptide (MSSSSWLLLSLVAVTAAQ) and a C-terminal V5-tag (GKPIPNPLLGLDST) together with a blasticidin-S-deaminase gene within the same cistron behind an ECMV IRES element. ADAM17 was amplified using Q5 polymerase and oligonucleotides ADAM17-deltaSP_forw (5′-GTAACTGCTGCTCAGTCCCCGCGACCGCCGGACGAG-3′) as well as ADAM17-V5_rev (5′-AGGGATAGGCTTACCGCACTCCGTTTCCTTGCTGTC-3′). The pEGIP-Blast vector backbone was amplified using Q5 polymerase and oligonucleotides pEGIP-SP_ rev (5′-CTGAGCAGCAGTTACAGCAACAAG-3′) and pEGIP-V5_forw (5′-GGTAAGCCTATCCCTAACCCTC-3′). An in vitro plasmid assembly reaction (NEBuilder) generated the plasmid pEGIP-Blast-ADAM17 (encoding amino acids 19–832 of porcine ADAM17). In order to prevent the unwanted cleavage of the recombinant gene by CRISPR/Cas9 after chromosomal integration, the sgRNA target sequence within the expression vector was mutated using the oligonucleotides ADAM17-guide-exchange_forw (5′-AGCATCAGACAGCACAGCGTGCGTAAAAGGGATCTGCAGGCCTCAAC-3′) and ADAM17-guide-exchange_rev (5′-GTGCTGTCTGATGCTGGATAAAGAGAGGATGTCGTAG-3′) without changing the encoded amino acids. The novel sequence (5′-GCATCAGACAGCACAGCGTGCGT-3′) resulted in a total of 8 nucleotide mismatches to the gRNA, effectively preventing recognition. Plasmid sequences were verified using full plasmid sequencing (Full PlasmidSeq; Microsynth, Balgach, Switzerland).

### 2.8. Lentiviral Transduction

Lentiviral pseudoparticles were produced using a commercial system (ViraPower; Thermo Fisher Scientific) for packaging and the pEGIP [67] and pLentiCRISPRv2 [66] vectors as compatible expression plasmids. Briefly, 293T cells were transfected using PEI (Polysciences Europe GmbH, Hirschberg, Germany) with plasmid DNA from the respective expression plasmid together with pLP1, pLP2, and pVSVG from the ViraPower Kit. The cells were incubated for 3 days before the supernatant was harvested and sterile filtered (Millex-HV Syringe Filter Unit—0.45 µm; Merck, Darmstadt, Germany). SK-6 cells were incubated with the produced pseudoparticles for 24 h before the supernatant was replaced by fresh medium. At day 3 after transduction, the cells were harvested by trypsinization and selected with puromycin (1 µg/mL) or blasticidin (3 µg/mL) for three days. Single-cell clones were generated by serial dilution under constant selection pressure. The successful knockout was tested by RT-PCR as described above. The antigen expression of the knock-in cell clones was verified by immunofluorescence tests using the V5-tag antibody (R960-25; Thermofisher). Validated cell clones were further expanded, cryopreserved, and used for the experiments.

### 2.9. Statistical Analysis

Differences in the means of data were compared using unpaired, two-tailed Student’s *t* tests. ANOVA was used when comparing multiple values. Significant differences were assumed for *p* values of <0.05. All statistical analyses were performed using the GraphPad Prism software (version 7.0d; GraphPad, San Diego, CA, USA).

## 3. Results

### 3.1. Pre-Infection of MDBK and SK-6 Cells with Different Pestiviruses

Cultures of MDBK and SK-6 cells had to be completely infected with one pestivirus species to reliably quantify the superinfection rates of other pestiviruses. It quickly became clear that certain virus–cell combinations did not result in sufficient virus spread, since there was probably a mismatch between the viral ligands and host cell receptors. To test the infection rate, aliquots of pre-infected cells were fixed, stained with the appropriate antibodies together with a nuclear counterstain (DAPI), and assessed with a fluorescence microscope. BVDV-1, BVDV-2, and HoBiPeV (BVDV-3) viruses infected SK-6 cells only very inefficiently and therefore failed to produce completely infected cultures. The pronounced species-specific host cell tropism of BVDV viruses had already been demonstrated in earlier studies [37,68]. Successful virus infections in SK-6 were established for APPV, BDV, BuPV, CSFV, and Linda virus (Figure S1). It has to be noted that the APPV-infected cells were generated by electroporation of synthetic RNA, as previously reported [57], because the field virus strain infections showed limited infection, but no spread in cultured cells. However, the small amount of infectious virus produced after electroporation was sufficient to completely infect the transfected culture within three passages. It was not possible to establish a pre-infection of MDBK cells with APPV, as these cattle cells seem to be unsusceptible. Successful virus infections in MDBK were established for BDV, BuPV, BVDV-1, BVDV-2, CSFV, HoBi, and Linda virus (Figure S2).

### 3.2. Superinfection Exclusion Experiments

Five different pestiviruses (BDV, BuPV, CSFV, Linda, and cpCSFV-mCherry) were chosen to analyze the susceptibility of the pre-infected cells. In order to mimic the natural infection processes, only non-cytopathogenic viruses were used for these superinfection experiments. Superinfection was detected at the single-cell level using specific antibodies that only reacted with antigens of the superinfecting virus species. Not every virus could be tested on each of the pre-infected cell cultures because cross-reactions of the monoclonal antibodies occurred due to the close antigenetic relationships of some pestivirus species. In order to obtain data on a homologous superinfection, a recombinant cytopathogenic CSFV strain developed using genetic engineering methods was used, which expressed an additional reporter gene (cpCSFV-mCherry) for reliable differentiation [33]. It was therefore possible to distinguish between pre-infection with CSFV and superinfection with this cpCSFV-mCherry without a need for immunofluorescence staining. The titrations of pestiviruses on the different pre-infected cell lines showed different statistically significant reductions of the TCID_50_ compared to naïve cells. However, an exception was found in APPV-pre-infected SK-6 cells. Titers of superinfecting pestiviruses were increased on these cells compared to the naïve control cells, which is why these titrations are presented separately in the following sections.

#### 3.2.1. Superinfection Exclusion of BDV in Pre-Infected Cells

**Figure 1:**
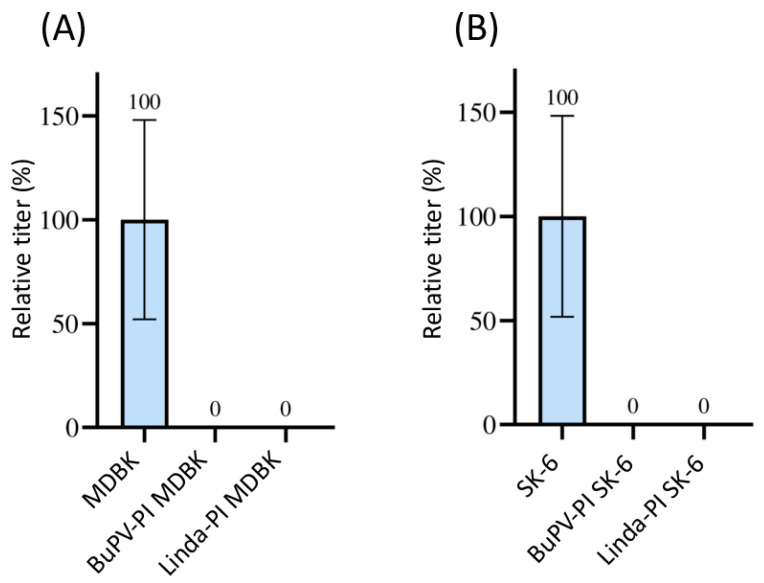
Superinfection exclusion of BDV in pre-infected cells. (**A**) Titration of BDV on naïve MDBK cells (blue) and on BuPV- as well as LindaV-pre-infected (PI) MDBK cells. (**B**) Titration of BDV on naïve SK-6 cells (blue) and on BuPV- and LindaV-pre-infected SK-6 cells. The mean value of the virus titer on the naïve cells was defined as 100% (TCID_50_ of 9.4 × 10^5^ on MDBK and 4.4 × 10^5^ on SK-6, respectively). The individual titers on the pre-infected cell cultures from three separated experiments were set in relation to these 100%. Titers were determined as TCID_50_ at 48 h p.i. The *Y*-axis shows TCID_50_ values in percent. The mean and standard deviation are depicted and the mean is indicated above each bar.

The mAbs 6A5 and 8.12.7, which were available for the detection of BDV strain X818 also reacted with the E2 or NS3 of CSFV, BVDV-1, BVDV-2, and HoBiPeV. Therefore, the effect on superinfection with BDV with pre-infections of these viruses could not be assessed. As only the novel pestivirus species BuPV and Linda virus could be clearly differentiated from BDV due to their lower antigenic similarities, these two pre-infections were tested. No foci of BDV-infected cells were observed after superinfection using 1 mL of supernatant containing 1 × 10^6^ TCID_50_ in the pre-infected cell lines (Figure 1). Hence, 100% superinfection exclusion was measured in all three titrations performed (*p* = 0.02). As BDV is a sheep pathogen, it should be noted that MDBK and SK-6 cells are somewhat disadvantageous host cells.

#### 3.2.2. Superinfection Exclusion of BuPV in Pre-Infected Cells

**Figure 2:**
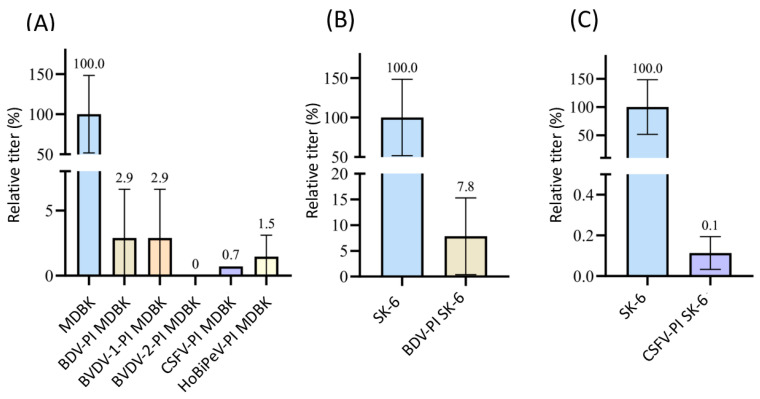
Superinfection exclusion of BuPV in pre-infected cells. (**A**) Titration of BuPV on naïve MDBK cells (blue), BDV, BVDV-1, BVDV-2, CSFV, and HoBi-PI cells (different colors). (**B**) Titration of BuPV on naïve SK-6 cells (blue) and BDV-PI SK-6 (light grey). (**C**) Titration of BuPV on naïve SK-6 cells (blue) and CSFV-PI SK-6 (violet). The mean value of the virus titers on the naïve cell lines was defined as 100% (TCID_50_ of 4.4 × 10^5^ on MDBK and 2.0 × 10^6^ on SK-6, respectively). The individual titers on the pre-infected cell cultures were set in relation to 100%. Evaluations were carried out 48 h p.i. The *Y*-axis shows TCID_50_ values as relative percentages and is shown in segments. The standard deviation was plotted. The calculated mean values in % are shown above the bars.

Since mAbs 11D5 anti NS3 solely reacted with BuPV and Linda virus, we were able to test BDV-, BVDV-1-, BVDV-2-, CSFV-, and HoBiPeV-pre-infected MDBK cells (Figure 2A). To investigate host cell-specific effects, BDV- and CSFV-pre-infected SK-6 cells were also included in the test (Figure 2B,C). The infections of BuPV in MDBK cells could be considerably limited by all pre-infections. It was striking that BVDV-2 (strain 890) caused complete superinfection exclusion (100%, *p* = 0.02), while BDV, BVDV-1, CSFV, and HoBi reduced the infection titer by more than one log level, still allowing focus formation (*p* < 0.05). Infections of BuPV were blocked with low efficiency by pre-infection of SK-6 cells with BDV, so that the superinfection exclusion rate was only 92.2% (*p* = 0.03). In contrast, pre-infection of SK-6 cells with CSFV was much more potent and reduced the infection titer of BuPV by two whole log levels (*p* = 0.02). The sheep virus BDV could only slightly block the pig virus BuPV on the porcine cells, which matches the reverse setup in which BuPV was able to completely prevent BDV infection of porcine cells. Similarly, CSFV, as a porcine virus, shows stronger blocking on pig cells than on bovine cells.

#### 3.2.3. Superinfection Exclusion of CSFV in Pre-Infected Cells

**Figure 3:**
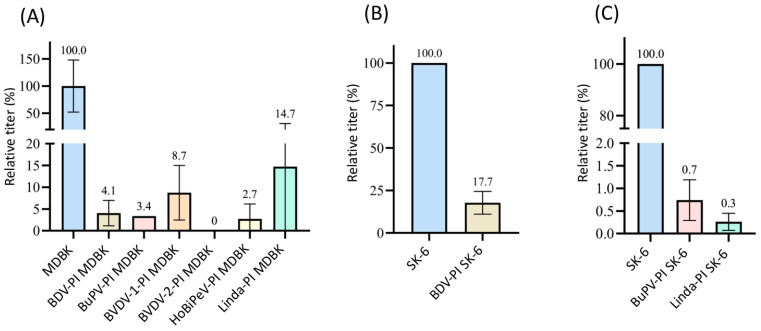
Superinfection exclusion of CSFV in pre-infected cells. (**A**) Titration of CSFV on naïve MDBK cells (blue) as well as BDV-, BuPV-, BVDV-1-, BVDV-2-, HoBiPeV-, and Linda virus-pre-infected cells (different colors). (**B**) Titration of CSFV on naïve SK-6 cells (blue) and BDV-PI SK-6 (light grey). (**C**) Titration of CSFV on naïve SK-6 cells (blue) and BuPV- (pink) as well as Linda-PI cells (mint green). The mean value of the virus titers on the naïve cells was defined as 100% (TCID_50_ of 9.4 × 10^3^ on MDBK and 3.2 × 10^5^ on SK-6, respectively). The mean titers on the pre-infected cell cultures were set in relation to 100%. The evaluation was carried out 48 h p.i. The *Y*-axis shows the TCID_50_ values as relative percentages and is shown in segments (**A**,**C**). The standard deviation is plotted and the calculated mean values in % are shown above the bars.

Since mAbs A18 anti E2 solely reacted with CSFV, we were able to test all other viruses included in this study. We found very different influences on the CSFV infection rates in pre-infected MDBK cells (Figure 3). Only BVDV-2-infected cells did not allow any superinfection (*p* = 0.02), while BDV-, BuPV-, and HoBiPeV-infected cells still measured 2.7 to 4.1% residual infection titers (*p* < 0.05). Surprisingly, the pre-infection of MDBK cells with BVDV-1 (8.7%) and Linda virus (14.7%) was very ineffective in blocking superinfection with CSFV (*p* < 0.04). SK-6 cells pre-infected with BDV, BuPV, and Linda were also included in the test (Figure 3B,C). While cells pre-infected with BDV still displayed 17.7% of the CSFV infection titer (*p* = 2.9 × 10^−5^), BuPV and Linda pre-infections blocked CSFV infection by more than 99% (*p* = 2.8 × 10^−10^ and *p* = 8.8 × 10^−12^).

**Figure 4:**
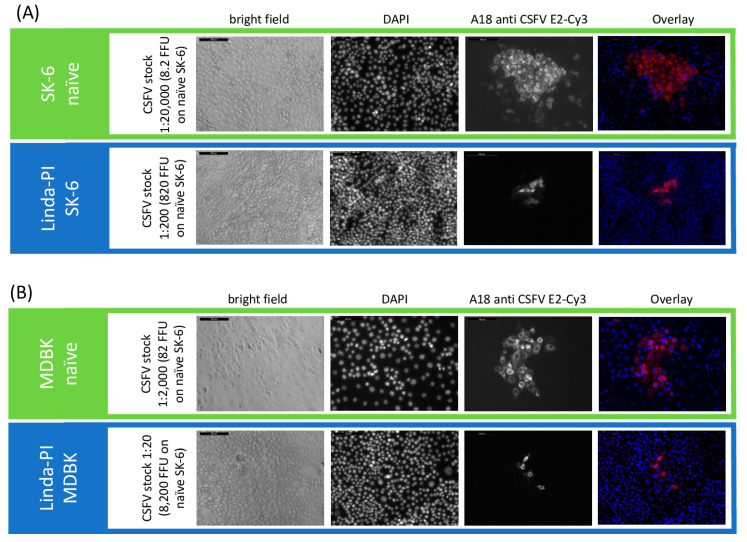
Superinfection exclusion reduces CSFV titer and focus size in Linda virus-pre-infected cells. A CSFV stock with a titer of 1.46 × 10^6^ TCID_50_/mL (8.2 × 10^5^ FFU/mL) was tested on (**A**) naïve and Linda virus-pre-infected SK-6 cells, as well as (**B**) naïve and Linda virus-pre-infected MDBK cells. The left side of the figure indicates the cell line, the CSFV stock dilution, and the FFU in the inoculum. Infection outcomes were analyzed 48 h p.i. using immunofluorescence. CSFV was detected with the E2-specific antibody A18 and a Cy3-conjugated secondary antibody. DAPI was used to stain cell nuclei, and the images were overlaid to analyze infection at the single-cell level. Images were captured at 10× magnification with a 200 μm scale bar included (top left). Notably, pre-infection with Linda virus not only restricted the primary infection but also reduced cell-to-cell spread, as evidenced by the smaller focus size.

The direct comparison of the titrations between the two cell lines (MDBK and SK-6 cells) from two different species shows that the superinfection exclusion between the cell lines does not occur to a comparable extent. While on Linda-PI MDBK cells only an exclusion of CSFV infection of approximately 85% occurred, Linda-PI SK-6 cells were practically protected from CSFV infections with an exclusion rate of >99%. These comparisons also show that BDV, as a sheep virus, in contrast to the swine viruses BuPV and Linda pestivirus, can hardly exert a blocking effect on the swine virus CSFV. The limited blocking effect of BVDV-1 on bovine cells, on the other hand, does not fit the picture. The effects of the superinfection exclusion could be observed in the experiments not only by the reduction of the observed titers in pre-infected cell lines but also by the significant effects on the formation and size of viral foci stained. As an example, the focus size was reduced in the case of CSFV on MDBK cells pre-infected with Linda virus to 4.2 cells per focus (SD = 1.5) in comparison to 43.2 cells per focus (SD = 9.2) on naïve MDBK cells. Hence, pre-infections impair not only the primary infection by free virus particles but also the spread of the virus infection from the infected to the neighboring cells (Figure 4). Interestingly, the percentage reduction in the focus size nicely corresponded to the percentage reduction in titer. Since the immunofluorescence signal in naïve cells and pre-infected cells had a comparable intensity, we found no indication of severely reduced replication rates or protein expression rates after successful superinfections.

#### 3.2.4. Superinfection Exclusion of Linda Virus in Pre-Infected Cells

**Figure 5:**
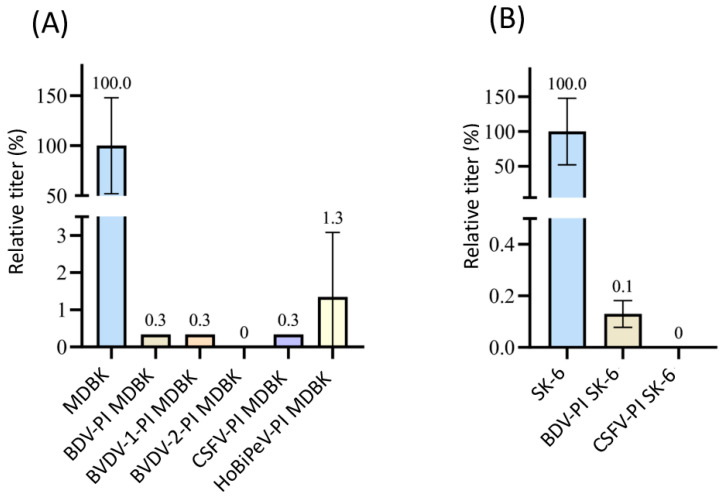
Superinfection exclusion of Linda virus in pre-infected cells. (**A**) Titration of Linda virus on naïve MDBK cells (blue), BDV-, BVDV-1-, BVDV-2-, CSFV-, and HoBiPeV-PI MDBK cells (different colors). (**B**) Titration of Linda virus on naïve SK-6 cells (blue), BDV- (light grey), and CSFV-PI SK-6 cells. The mean value of the virus titers on the naïve cells was defined as 100% (TCID_50_ of 9.4 × 10^4^ on MDBK and 9.4 × 10^5^ on SK-6, respectively). The titers on the pre-infected cell cultures were set in relation to 100%. Evaluation was carried out 48 h p.i. The *Y*-axis shows the TCID_50_ values as relative percentages and is shown in segments. The standard deviation was plotted and mean values in % are shown above the bars.

For the Linda virus, we were able to use the 11D5 antibody and were thus able to test BDV-, BVDV-1-, BVDV-2-, CSFV-, and HoBiPeV-pre-infected MDBK cells. (Figure 5). Apart from the cells infected with HoBi, which showed 98.7% superinfection exclusion, pre-infection with the other pestiviruses almost completely prevented infection with Linda virus (residual infectivity of maximum 0.3%, *p* < 0.05). As with almost all superinfection tests performed, an exclusion rate of 100% (*p* = 0.02) was measured for BVDV-2 PI MDBK cells. Superinfection with Linda led to an exclusion rate of over 99% on BDV-PI SK-6 cells (*p* = 0.02) and 100% on CSFV-PI SK-6 cells (*p* = 0.02). Although Linda pestivirus has been isolated from diseased pigs, a spillover infection seems likely, as no pig reservoir has yet been identified [69]. It is therefore quite possible that neither bovine cells nor pig cells are optimal host cells for Linda virus, so that even the sheep virus BDV causes a strong block in porcine cells.

#### 3.2.5. Increase in Susceptibility of SK-6 Cells Due to APPV Pre-Infection

**Figure 6:**
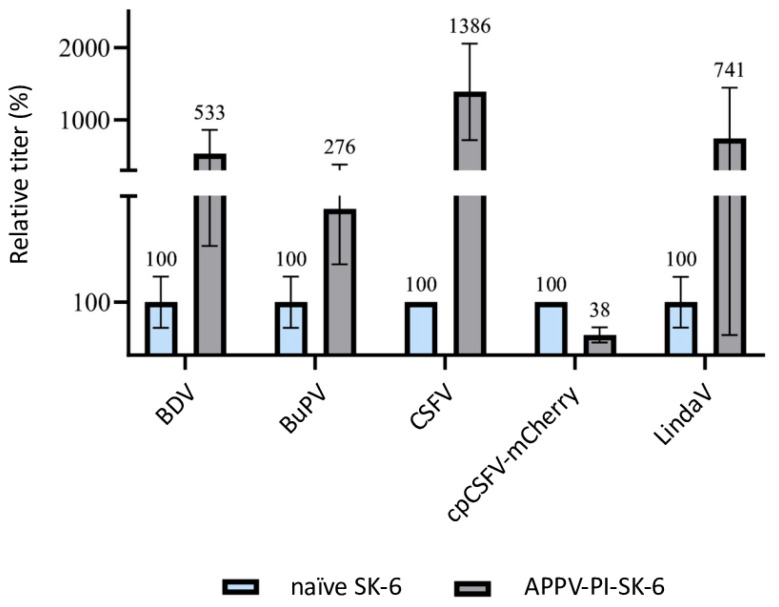
Superinfection enhancement in APPV-pre-infected SK-6 cells. Titration of BDV, BuPV, CSFV, cpCSFV-mCherry, and Linda on naïve SK-6 cells (each shown in blue) and APPV-PI SK-6 cells (each shown in gray). Evaluation was carried out 48 h after infection by determining the TCID_50_. The mean values of the virus titers on the naïve SK-6 cells were defined as 100% (TCID_50_ on naïve SK-6 of 4.4 × 10^5^ for BDV, 2.0 × 10^6^ for BuPV, 3.2 × 10^5^ for CSFV, 1.5 × 10^6^ for cpCSFV, and 9.4 × 10^5^ for LindaV). The mean titers on the pre-infected cell cultures were set in relation to the 100%. The *Y*-axis shows the relative percentages and is shown in segments. The standard deviation is plotted and the calculated mean values in % are shown above all bars.

**Figure 7:**
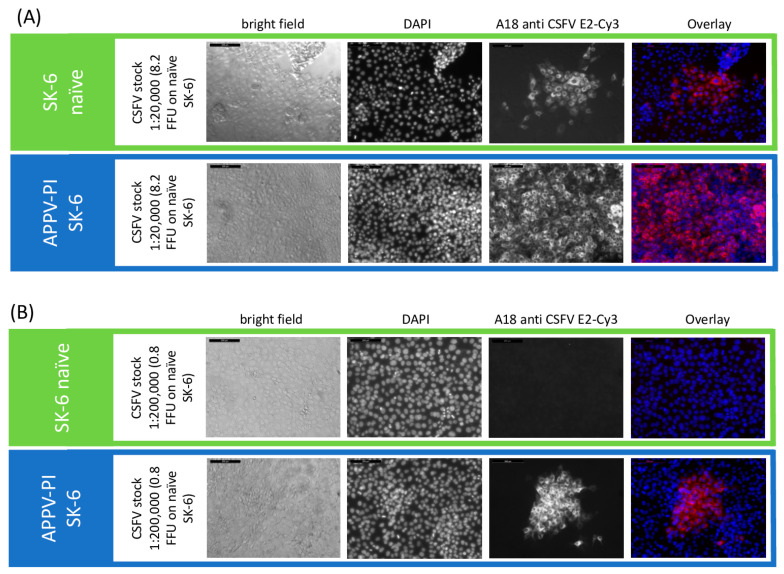
Enhancement of superinfection by APPV does not alter plaque size. Titrations of CSFV were tested on naïve SK-6 cells and APPV-pre-infected SK-6 cells using (**A**) a virus stock dilution of 1:20,000 and (**B**) of 1:200,000. CSFV was detected with the E2-specific antibody A18. A goat anti-mouse IgG Cy3 conjugate was used to visualize primary antibody binding. Nuclei were stained with DAPI to determine the localization of individual cells and the two fluorescence channels were superimposed (overlay). Images were captured at 10× magnification and a 200 μm bar is shown. The dilution of 1:20,000 produced single foci on naïve cells but resulted in a complete infection of the monolayer in the APPV-pre-infected cells. (**B**) The virus stock dilution of 1:200,000 resulted in no infection of naïve SK-6 cells but still yielded foci on APPV-pre-infected cells. Note the similar focus size on both cell lines despite the different apparent virus titers.

Surprisingly, when performing the same experiments with APPV-PI SK-6 cells, an increase in the titer of superinfecting viruses was observed (Figure 6). APPV is only very distantly related to the other pestiviruses and its proteins do not cross-react with the monoclonal antibodies used to detect the other pestiviruses. Increases in the titers of the viruses BDV (*p* = 0.09), BuPV (*p* = 0.2), CSFV (*p* = 0.03), and Linda (*p* = 0.19) occurred compared to the respective naïve controls. Only the titer of cpCSFV-mCherry showed a decrease (superinfection exclusion) but with residual values of >35% (*p* = 0.002). Enhancing effects of pre-infections are so far unknown between pestiviruses. To evaluate whether the spread of the superinfecting virus is increased in APPV-infected cells, which would indicate an improved replication, particle production or cell-to-cell spread, we compared the focus size of CSFV after immunofluorescence staining (Figure 7). However, despite the higher infection dose of the inoculum, we found no apparent change in focal size or morphology. In a side-by-side comparison, CSFV reached focus sizes with an average of 119.4 cells per focus (SD = 12.7) in naïve SK-6 cells within 48 h p.i. and foci with an average size of 124.4 cells per focus (SD = 21.4) on the APPV-pre-infected SK-6 cells. Given the low infectivity of our wild-type APPV and the slow spread of our cell culture-adapted APPV isolates, we were not able to reliably quantify the effects of pre-infections with classical pestiviruses on APPV titers. However, we did not observe the spread of the wild type APPV on cells that were pre-infected with CSFV.

#### 3.2.6. Superinfection Exclusion of cpCSFV-mCherry in Pre-Infected Cells

**Figure 8:**
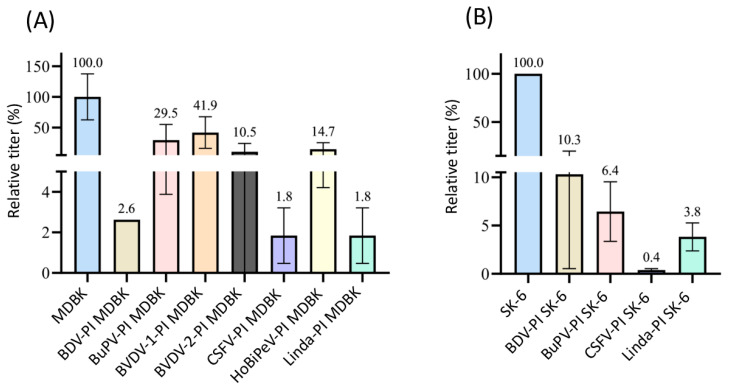
Superinfection exclusion of cpCSFV-mCherry in pre-infected cells. (**A**) Titration of cpCSFV-mCherry on naïve MDBK cells (blue), BDV-, BuPV-, BVDV-1-, BVDV-2-, CSFV-, HoBiPeV-, and Linda virus-pre-infected MDBK cells (different colors). (**B**) Titration of cpCSFV-mCherry on naïve SK-6 cells (blue), as well as BDV-, BuPV-, CSFV-, and Linda virus-pre-infected SK-6 cells. The mean value of the virus titers on the naïve cells was defined as 100% (TCID_50_ of 1.2 × 10^4^ on MDBK and 1.5 × 10^6^ on SK-6, respectively). The individual titers on the pre-infected cell cultures were set in relation to 100%. Evaluation was carried out 48 h p.i. The *Y*-axis shows the TCID_50_ values as relative percentages and is shown in segments. The standard deviation is plotted and the calculated mean values in % are shown above the bars.

In addition to the influence of the expression of viral structural proteins, RNA replication interferences have been found to be another reason for the exclusion of superinfection in pestiviruses [14]. Since these studies were performed using cp pestiviruses, we also wanted to test a cp virus with a significantly higher replication level than ncp pestiviruses to see whether this effect also becomes apparent in the experimental setup used. To measure the efficacy of superinfection with an identical virus, we used a genetically modified cpCSFV strain carrying an mCherry reporter gene. Although plaque formation in such cp pestiviruses is usually rather slow, this approach allowed a precise quantification of the superinfections (Figure 8).

In agreement with existing data on the influence of viral replication on superinfection exclusion, our titrations of cpCSFV-mCherry showed a significant lower superinfection exclusion rate in direct comparison with the ncp CSFV. Although in the case of BDV-, CSFV-, and Linda-PI MDBK cells, where still more than 95% (in all cases *p* = 0.01) of the infection events were blocked, only low superinfection exclusions rates occurred on BuPV- and BVDV-1-PI MDBK (70% and 59%, respectively), also meaning that no significant difference was found against naïve cells (*p* > 0.05). Titration for BVDV-2-PI MDBK cells also did not lead to the complete exclusion of the cpCSFV infection, but only resulted in a rate of around 90% (*p* = 0.02). Pre-infection of SK-6 cells from the original host species resulted in about 90% superinfection exclusions in most combinations. However, the titration of cpCSFV-mCherry on CSFV-PI SK-6 cells resulted in a higher exclusion rate of over 99% (*p* = 2.9 × 10^−12^). This indicates that superinfection exclusion had the strongest effect against the homologous virus species, whereby in addition to the inhibition of entry, an influence of replication interference must also be taken into account. These genetically homologous viruses certainly require the same host factors.

### 3.3. ADAM17 Knockout Can Abolish and Knock-In Can Restore Susceptibility of SK-6 to cpCSFV-mCherry

ADAM17 has recently been described as an essential attachment factor for CSFV E2 and this finding was extended to other pestiviruses including BVDVs [46,47]. Hence, we wanted to investigate whether superinfection exclusions between classical pestiviruses and superinfection enhancement in APPV-infected cells might be correlated with ADAM17–E2 interactions. For this purpose, we generated ADAM17 knockout SK-6 cells, which were validated by RT-PCR allele sequencing. These cells nicely confirmed the previous data of Yuan et al., as the ADAM17 knockout SK-6 cells were completely resistant to CSFV infection. After subsequent knock-in of the ADAM17 gene with a mutated guide sequence (Figure S3), the cells regained susceptibility to CSFV infections (Figure S4). To detect the recombinant ADAM17 expression, a V5 tag was used, which was inserted at the C-terminus of ADAM17 enabling detection in immunofluorescence and Western blot tests, although no porcine ADAM17-specific reagents were available.

### 3.4. APPV Infection Is Independent of ADAM17

**Figure 9:**
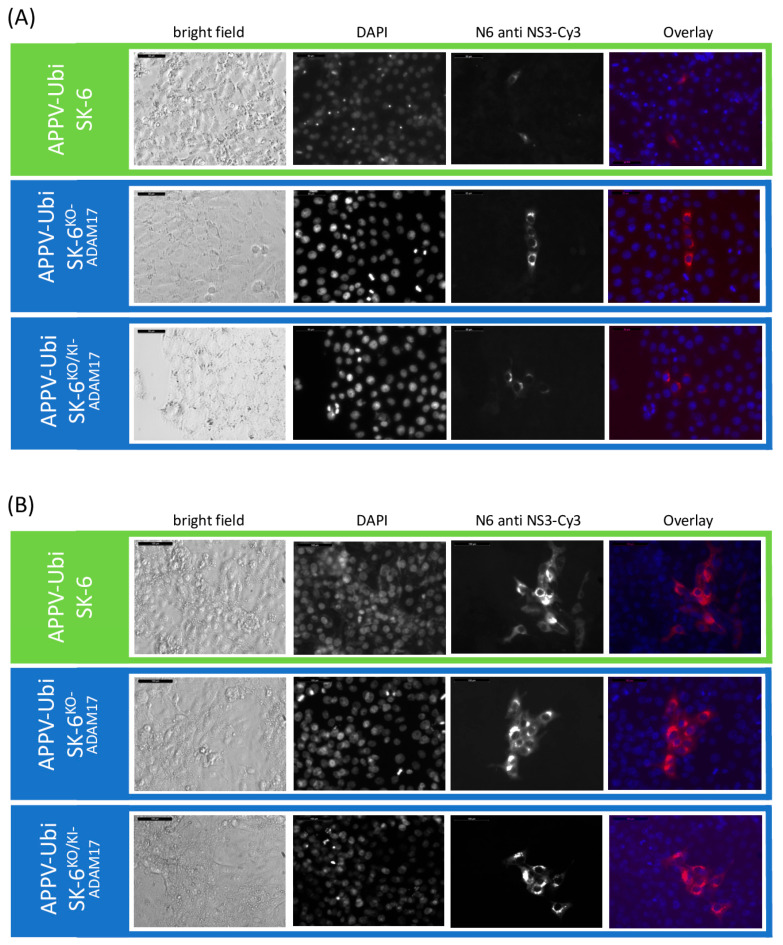
APPV-Ubi infections are independent of the expression of ADAM17. Infection of mock treated with APPV-Ubi. At 24 h (**A**) and at 48 h (**B**) p.i., the APPV-Ubi infections of SK-6 cells, SK-6^KO-ADAM17^, and SK-6^KO/KI-ADAM17^ were evaluated via immunofluorescence staining using antibody N6. The cells were fixed with PFA, immunostained, and counterstained with DAPI. The Cy3 fluorescence and the nuclear DAPI signals were recorded and both images were merged (overlay). A brightfield image was made for comparison. Images were taken at 32× magnification at 24 h and at 20× magnification at 48 h including a scale bar (50 µm and 100 µm, respectively). Note the similar growth of the APPV-Ubi on all three cell lines.

We used a similar setup to test the susceptibility of ADAM17 knockout and knock-in cells to APPV. Since field strains of APPV do not readily spread in cultured cells, we utilized a genetically modified APPV strain containing duplicated NS3 to NS4B sequences (APPV-Ubi) [57], which was subsequently adapted to growth on SK-6 cells. The cell culture-adapted APPV-Ubi still yielded only low titers of about 5 × 10^2^ FFU/mL. However, this virus properly spreads from the infected cells resulting in larger foci of infected cells and ultimately complete infection of the monolayer. We found no differences in our susceptibility tests between the control SK-6 cells, the SK-6^KO-ADAM17^, and the SK-6^KO/KI-ADAM17^ cells (Figure 9). As early as 24 h post infection, infected cells became detectable in immunofluorescence staining across all cultures, irrespective of ADAM17 expression. After 48 h, foci of similar sizes occurred in all three cell lines. Due to the low titer and slow virus spread, the susceptibility of the cells to APPV-Ubi was not determined using TCID_50_ tests but rather as FFU/mL. The average of three titrations resulted in titers of 4.7 × 10^2^ FFU/mL (SD = 5.8 × 10^1^) on the SK-6 cells, 5.3 × 10^2^ FFU/mL (SD = 5.8 × 10^1^) on the SK-6^KO-ADAM17^ cells, and 4.0 × 10^2^ FFU/mL (SD = 1.0 × 10^2^) on the SK-6^KO/KI-ADAM17^ cells. The average size of the foci at 48 h post infection was 13.8 (SD = 5.7) on the SK-6 cells, 13.2 (SD = 5.6) on the SK-6^KO-ADAM17^ cells, and 12.7 (SD = 4.8) on the SK-6^KO/KI-ADAM17^ cells. No statistically significant differences were found between these titers and the focus sizes (*p* > 0.05). From these results, we conclude that our cell culture-adapted APPV strain is not dependent on ADAM17 as an attachment factor. Infection tests using a non-adapted APPV cDNA clone also resulted in single-infected SK-6^KO-ADAM17^ cells. As no subsequent viral spread was observed in any of the SK-6 cell lines, further analyses were not pursued with this wild-type APPV clone.

## 4. Discussion

In this study, we used ncp pestiviruses affecting livestock and two distinct cell lines from different host species to investigate virus and host species-specific traits of superinfection exclusion. Although we aimed to fully map superinfection exclusion across all classical pestiviruses, we encountered two primary challenges. First, not all viruses could productively infect the SK-6 or MDBK cells employed in our experimental setup. Pestivirus experts might suggest that single-cell cloning of infected cells, which is commonly used to establish persistently infected cultures, could have been applied for mismatched virus/host cell combinations. However, based on our own preliminary tests, we advise against using subcloned cells for susceptibility testing. Subcloning tends to produce variability in susceptibility, similar to what has been observed with clonal knockout cell lines, despite side-by-side generation and targeting of the same gene [70]. Given the ongoing discovery of new pestivirus species, the expanding host range, and the diversity of suitable host cells, the combinations are nearly limitless. Thus, we focused on well-characterized livestock pestiviruses of cloven-hooved animals and two well-characterized cell lines, which can be infected by many different viruses. Second, detecting superinfection events requires specific reagents to differentiate between the primary and superinfecting species. One possible approach would have been to use cp strains to bypass the need for antigen detection, allowing even superinfection tests with closely related viruses. However, not all pestiviruses have such cp variants, and our experiment with cpCSFV revealed significant differences compared to the ncp wildtype counterpart. Regarding differentiating antibodies, our research group has a wide collection of species-specific monoclonal antibodies targeting various pestivirus proteins and species. Unfortunately, many of these antibodies showed weak cross-reactivities, complicating the reliable analysis of superinfection events. We therefore restricted our study to highly specific antibodies already validated in the past.

**Figure 10:**
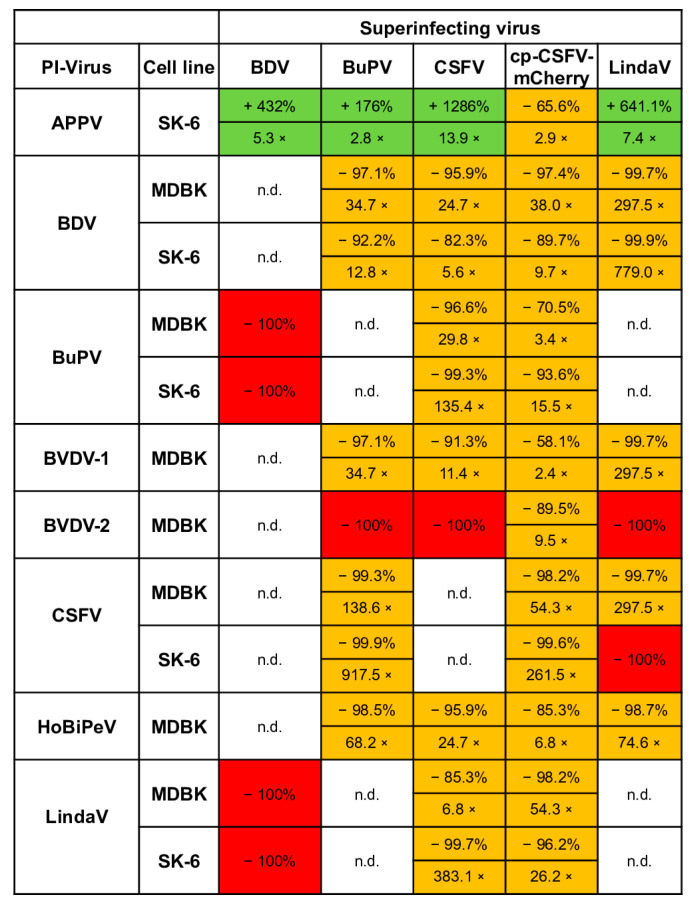
Synopsis of superinfection exclusion between pestiviruses. The table shows rates of superinfections of pestiviruses on pre-infected cells. Comparing naïve and pre-infected cells, we calculated the percentage of the apparent titers in the pre-infected cells as well as the reduction/increase factor. Titer increases are highlighted in green and reductions in orange. A complete superinfection exclusion was marked with a red field. The values were determined from three titrations separated in time and the mean values of the titers were subtracted from the corresponding controls. In the case of increases, the control was subtracted from the measured titer to indicate the effective rise. Combinations that were either not tested due to cross-reactivity of the mAbs or because the respective pre-infected cell lines could not be generated were labelled with n.d. (not done).

In this study, we assessed superinfection exclusion by comparing the reduction in apparent virus titers between pre-infected and naïve cells. The pre-infected cells were passaged until the experiments were completed. We must note that no weakening of the superinfection exclusion effect with the time of pre-infection was seen in our experiments, since the same virus stocks produced very similar titers even after 10 passages of the cells. This finding contrasts with the findings obtained using ncp/cp BVDV tests [14], but it must be noted that we did not investigate the early stages of pre-infection in this study. Hence, it is quite possible that even higher superinfection exclusions rates could have been observed in the first few hours after initial infections. In the setup chosen here, we had to use fully infected cultures, which is why all pre-infected cell lines were passaged at least three times and were therefore infected for more than 10 days before being included in our experiments. As complete superinfection exclusions were measured in multiple combinations, we cannot confirm that the phenomenon of superinfection exclusion is attenuated within 3 days and mostly lost after one passage, as previously reported [14]. Our results using different viruses and host cells demonstrate that superinfection exclusion in pestiviruses is complex and influenced by all key variables: the host cell line, the pre-infecting virus species, and the superinfecting virus species (Figure 10). Our tests showed in 31 out of 43 possible combinations an exclusion rate of 90% or above (fields colored in dark orange). In eight cases, the exclusion rate was below 90% (fields colored in light orange) and only the four APPV pre-infection tests showed an enhancement (fields colored in green). The picture of superinfection exclusion in our titrations was always similar showing decreased apparent titers on the pre-infected cells and smaller foci compared to the naïve cell lines, indicating a block of initial infections and of subsequent cell-to-cell spread. Conversely, however, no larger foci were found following the increase in infection titers on APPV-pre-infected SK-6 cells, indicating more efficient initial infections with subsequent “normal” cell-to-cell spread. We further found that a pestivirus is more effective at blocking a superinfecting virus in a well-suited host cell, particularly when that host cell is suboptimal for the superinfecting virus. For instance, BVDV-1 pre-infection reduced Linda virus titers by over 99% in MDBK cells, whereas BDV pre-infection in pig cells reduced CSFV titers by just 82%. It seems a plausible explanation that CSFV, a virus normally found in pigs, has a competitive advantage over BDV due to its better adaptation to pig cells. Since this competitive advantage of CSFV can only be observed in cells of its own target species, stronger affinities to target molecules such as receptors or replication factors could play a role. Beyond host cell suitability, viral replication levels significantly impact superinfection outcomes. Since replication rates vary even among strains of the same species, this phenomenon is also strain-specific rather than only species-specific. For example, BVDV-2 strain 890, with its high replication rates [71], completely excluded infections with other ncp pestiviruses. However, it was outcompeted by the cpCSFV, which can achieve much higher replication levels than an ncp pestivirus [33].

It is known that all stages of the viral infection cycle—attachment, entry, uncoating, translation, and genome replication—may be subject to interference. Previous studies on BVDV linked superinfection exclusion in pestiviruses to E2 expression, indicating that E2 may block or regulate as yet unknown host entry factors [14]. Current investigations focused on ADAM17 and documented a downregulation of mature ADAM17 on the cell surface following E2 expression of BVDV and CSFV, but not of LindaV or APPV E2 [72]. However, the precise role and mechanism in superinfection exclusion remains inconclusive, as elevated or reduced ADAM17 levels were not consistently associated with cellular susceptibility of MDBK cell mutants. Important cellular factors that determine pestivirus host specificity are still unknown but likely of importance for the dependence of superinfection exclusion on the host cell species and the pre-infecting virus species. While there is no evidence that translation is impaired by pre-infections, the expression of NS2-3 and viral genome replication is contributing to interference, as newly arriving viruses compete for limited cellular resources. DNAJC14, essential for NS2-3 processing in ncp pestiviruses, likely plays a role here. Cp pestiviruses that do not rely on DNAJC14 are not as strongly blocked [32] and APPV, which also does not require DNAJC14, does not induce superinfection exclusion [57].

The most surprising outcome of our study was certainly that pre-infecting SK-6 cells with APPV increased the apparent titers of classical pestiviruses. For CSFV, the titer in APPV-pre-infected cells even rose more than tenfold, indicating no block or interference related to receptor usage or competition for replication factors between these viruses. In a previous study, we demonstrated that APPV does not utilize the host cell factor DNAJC14 [57], which is critical for the other ncp pestiviruses. In the present study, we demonstrate using knockout cells that APPV, unlike classical pestiviruses, does not engage the essential attachment factor ADAM17, eliminating interference from this pathway as well. This can be interpreted as an indirect explanation for the lack of superinfection exclusion and as evidence for a crucial role of E2-ADAM17 interactions in mediating superinfection exclusion among classical pestiviruses. However, this does not explain the observed increase in infectivity of classical pestiviruses. One possibility is that the cell culture models used may only be partially susceptible to infection and frequently block the early stages of pestivirus replication. Again, all steps of the viral life cycle should be considered. Pre-infection with APPV might elevate the expression of certain surface attachment and entry factors, thereby enhancing initial attachment and entry processes. Similarly, the replication of classical pestiviruses could directly benefit from APPV protein expression, as APPV-induced membrane remodeling or potential interactions between APPV replicase proteins and classical pestivirus genomes could create a more favorable environment. Another explanation might be the presence of known pestiviral virulence factors. APPV expresses a potent N^pro^ protein that degrades IRF3, a key innate immunity factor [73]. It also harbors an effective E^rns^ protein that degrades immune stimuli acting as an RNase [74]. Thus, APPV pre-infection could suppress the innate immune response against a superinfecting virus and thereby enable more successful infection events. Historically, a superinfection phenomenon termed exaltation of Newcastle disease virus (END) was used to detect CSFV and BVDV infection of cultured cells [75]. Cytopathic effects emerged, when the typically ncp Newcastle disease virus infected culture cells already harboring ncp pestiviruses. The diagnostic value of END-tests was limited by the identification of END-phenomenon-negative virus strains. It was later demonstrated that the induction of type I interferon response by IRF-3 (END−), or the inhibition of this response by N^pro^-mediated degradation of IRF-3 (END+), is responsible for cytopathic effect occurrence [76]. However, in case of APPV a further investigation of the enhancement of the infection phenomenon is warranted, as this finding could have technical implications for the diagnosis and analysis of pestiviral diseases. Virus isolation in acute cases of BVDV and BDV is often challenging but essential for epidemiological tracking through genomic pathogen sequencing. The same applies to the isolation of CSFV from dead and rotting wild boar carcasses. Understanding the mechanisms behind superinfection enhancement and optimizing host cell susceptibility could simplify diagnostics and improve control of these notifiable diseases.

Overall, our study highlights the need to consider both host and viral factors to fully unravel the complexities of superinfection exclusion and enhancement in pestiviruses. Despite significant advances in recent decades, many aspects of pestiviral attachment, entry, and replication initiation remain obscure, warranting continued research in this field.

## Figures and Tables

**Table 1:** Antibodies used in this study.

Designation of the Hybridoma Cell Line and of the Monoclonal Murine Antibody	Specificity of the Serological Reagents	References
A18	E2 protein of CSFV	[63]
6A5	E2 of pestiviruses (BVDV-1, BVDV-2, BVDV-3, BDV, Bungowannah virus, and Linda virus)—weak reactivity with E2 of CSFV	[61,64]
N6	NS3 protein of APPV	[57]
8.12.7 (Code 4)	NS3 protein of classical pestiviruses (BDV, BVDV-1, -2, -3, CSFV)	[65]
11D5	NS3 protein of Bungowannah virus and Linda virus	[61]
R960-25	V5 tag	Novex

## Data Availability

All data analyzed or generated during this study are included in the manuscript.

## Supplementary Materials

The following supporting information can be downloaded at: https://www.mdpi.com/article/10.3390/v16121834/s1. Figure S1: Immunofluorescence test of pre-infected SK-6 cell cultures; Figure S2: Immunofluorescence test of pre-infected MDBK cell cultures; Figure S3: Expression of ADAM17 in SK-6^KO/KI-ADAM17^ cells; Figure S4: Nuclear expression of ADAM17 mRNA can compensate for infection blockade of CRISPR/Cas9-mediated knockout of ADAM17.
