# Supplementary material for: Exclusion of Superinfection or Enhancement of Superinfection in Pestiviruses—APPV Infection Is Not Dependent on ADAM17
Source: Viruses. 2024 Nov 26;16(12):1834. doi: 10.3390/v16121834 (PMC11680174; doi:10.3390/v16121834)
Supplement: Supplementary file 1 [file viruses-16-01834-s001.zip › viruses-3313490-supplementary-revised.pdf]

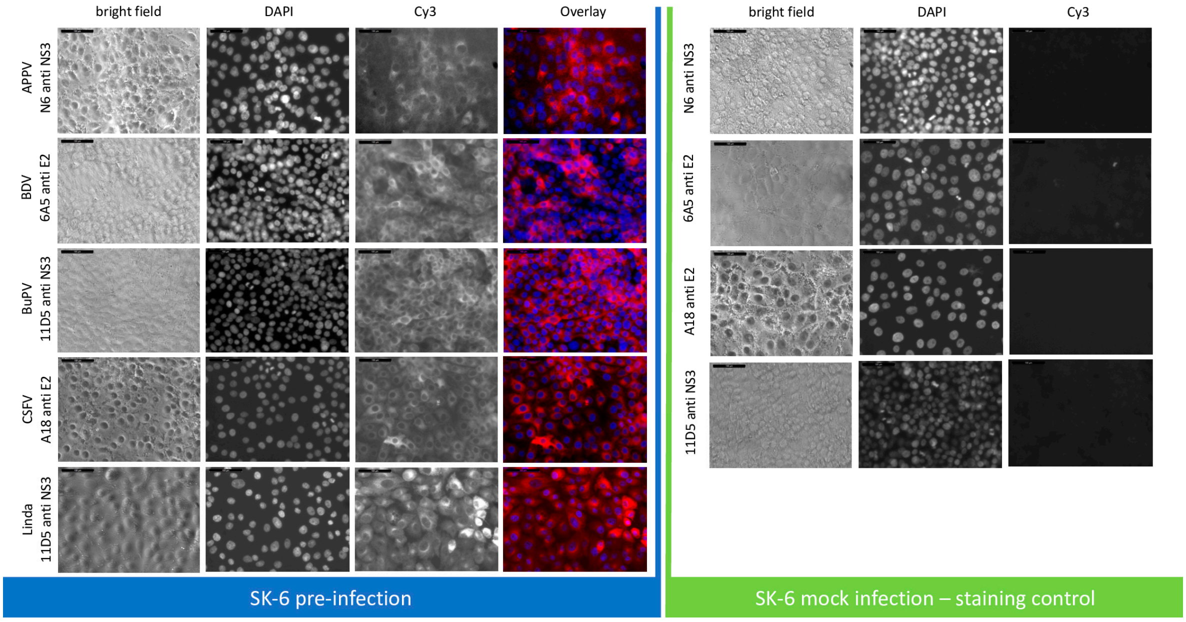

Figure S1: Immunofluorescence test of pre-infected SK-6 cell cultures. After three passages following the initial infection, a subculture was immunostained with a pestivirus-specific antibody. APPV was stained with the NS3-specific antibody N6, BDV with the E2-specific antibody 6A5, and CSFV with the E2-specific antibody A18. BuPV and LindaV were stained with the NS3-specific antibody 11D5. A Cy3 conjugate of goat anti-mouse IgG was used to visualize the binding of the primary antibodies. The cellular nuclei were stained with DAPI to determine the localization of individual cells. The left column shows bright field images, followed by DAPI stain, Cy3 immunofluorescence signal, and the overlay of DAPI stain (blue) and Cy3 signals (red) for the pre-infected cultures. The right column shows the staining controls of naïve SK-6 cells for each antibody used. Images were captured at 20x magnification with a 100 µm size marker (top left).

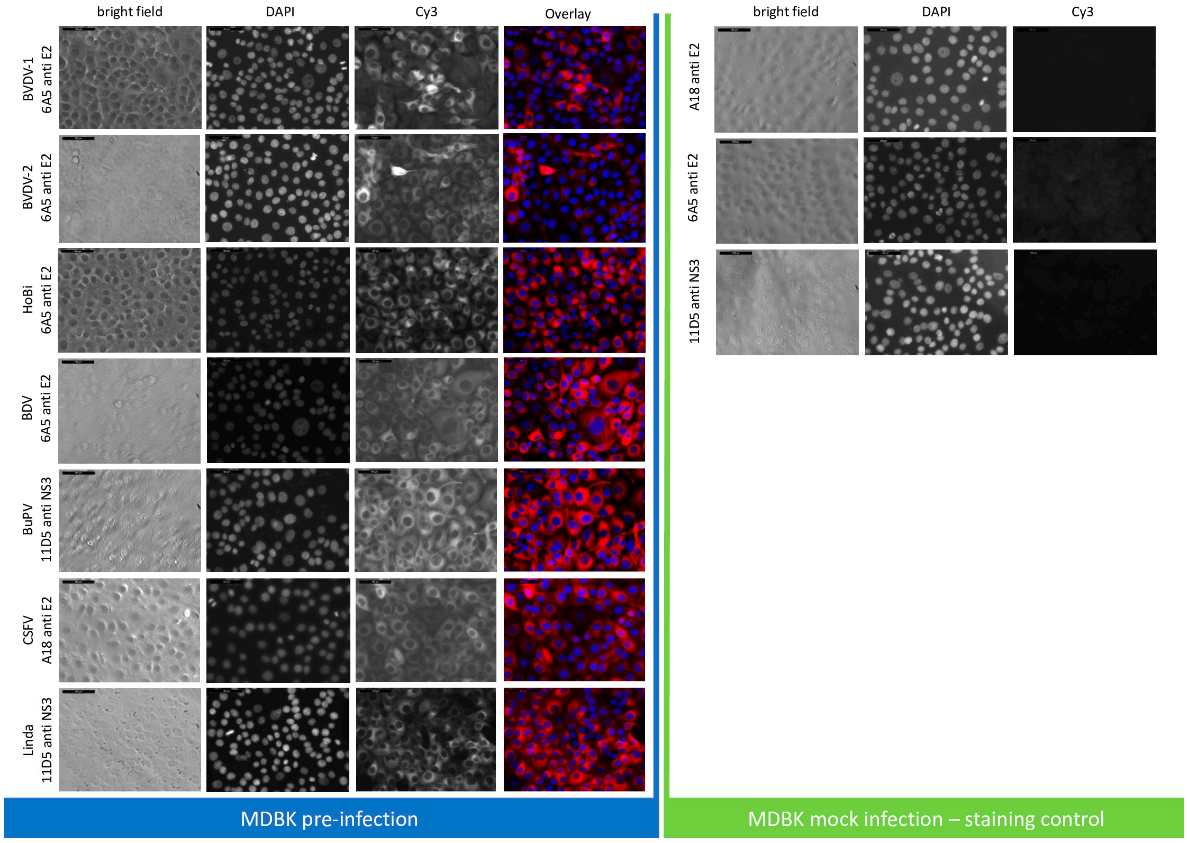

Figure S2: Immunofluorescence test of pre-infected MDBK cell cultures. After three passages following the initial infection, a subculture was immunostained with pestivirus-specific antibodies. BDV, BVDV-1, BVDV-2 and HoBiPeV were stained with the E2-specific antibody 6A5. CSFV was stained with the E2-specific antibody A18. BuPV and LindaV were stained with the NS3-specific antibody 11D5. A Cy3 conjugate of goat anti-mouse IgG was used to visualize the binding of the primary antibodies. The cellular nuclei were stained with DAPI to determine the localization of individual cells. The left column shows bright field images, followed by DAPI stain, Cy3 immunofluorescence signal, and an overlay of DAPI stain (blue) and Cy3 signals (red). A negative control staining of naïve MDBK cells with the respective antibodies is presented at the right side. Images were captured at 20x magnification with a 100 µm size marker (top left).

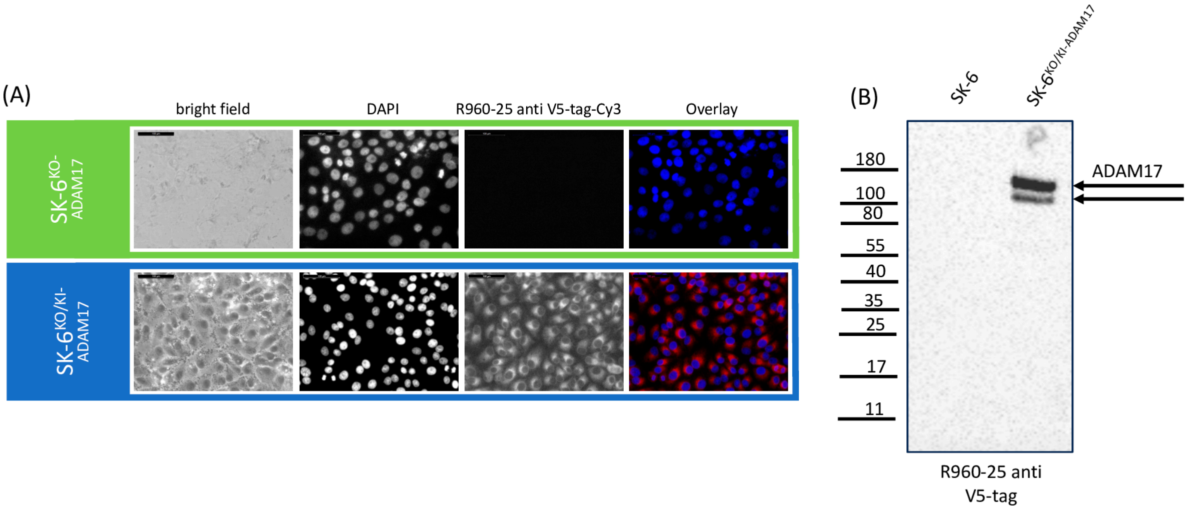

Figure S3: Expression of ADAM17-V5 in SK-6$^{KO/KI-ADAM17}$ cells. (A) Immunostaining of SK-6$^{KO-ADAM17}$ cells and a blasticidin selected SK-6$^{KO/KI-ADAM17}$ cell clone. The cells were fixed with PFA and stained using the V5-tag-specific antibody R960-25. A goat anti-mouse IgG Cy3 conjugate served as a secondary reagent. Nuclei were stained with DAPI and both stainings were merged (overlay). A brightfield image was also made for comparison. Images were taken at 20x magnification including a scale bar (100 µm). Note the homogenous expression pattern of ADAM17. (B) Westernblot analysis of non-transduced SK-6 cells and SK-6$^{KO/KI-ADAM17}$ cells. A strong expression of the proenzyme (calculated unglycosylated molecular weight of 93 kDa, apparent molecular weight about 120 kDa) and a weaker band of the mature protease (calculated unglycosylated molecular weight 69 kDa, apparent molecular weight about 100 kDa) is detected.

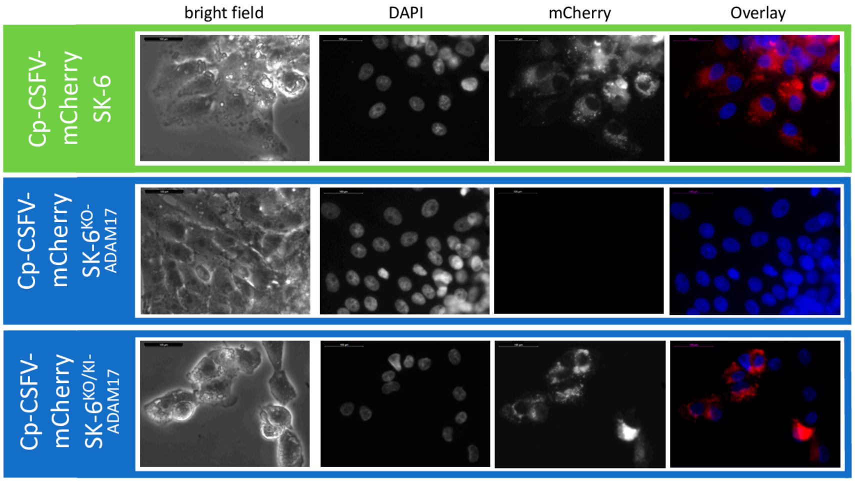

Figure S4: Knock-in of the porcine ADAM17 gene re-establishes susceptibility of ADAM17 knockout cells to CSFV. Infection of mock treated SK-6 cells, SK-6$^{KO-ADAM17}$ and SK-6$^{KO/KI-ADAM17}$ with cpCSFV-mCherry. Two days after inoculation, the CSFV infections were evaluated using the reporter gene. Therefore, the cells were fixed with PFA and stained with DAPI. The mCherry fluorescence and the nuclear DAPI signals were recorded and both images were merged (overlay). A brightfield image was also made for comparison. Images were taken at 20x magnification including a scale bar (100 µm). Note the strong cytopathic effects on the cells caused by the infection.
